# Bridging institutional expertise and democratic decentralization: a proposed hub-and-spoke framework for rehabilitation, built on Kerala’s model of community-based disability care

**DOI:** 10.3389/fresc.2026.1927112

**Published:** 2026-10-08

**Authors:** Binoy Mathew K V, Maryelizabeth Tidiya Walarine

**Affiliations:** 1 Department of Physiotherapy, Composite Regional Centre for Skill Development, Rehabilitation and Empowerment of Persons with Disabilities (CRC), Kozhikode, Kozhikode, Kerala, India; 2 Department of Medical Surgical Nursing, MIMS College of Nursing, Malappuram, India

**Keywords:** community-based rehabilitation, decentralization, disability, health systems strengthening, hub-and-spoke model, Kerala, Rehabilitation 2030, task-sharing

## Abstract

Community-based rehabilitation (CBR) is the World Health Organization's (WHO) endorsed strategy for extending disability services in low- and middle-income countries (LMICs), yet documented implementation problems — workforce shortage, fragmented supervision, and weak evidence on long-term adherence — continue to limit its reach, while centralized specialist institutes remain constrained by travel-related attrition among the families they serve. Kerala, India, has built one of the more mature decentralized CBR infrastructures in an LMIC setting: Local Self-Government Institutions (LSGIs), acting jointly with Kudumbashree (a state-wide community network of women) and drawing on their statutory healthcare responsibilities toward persons with disabilities under Section 25 of India's Rights of Persons with Disabilities (RPwD) Act 2016, fund and operate close to 330 BUDS Schools and BUDS Rehabilitation Centres (BRCs) that already provide neighbourhood-level day-care, basic special education, and vocational support. This existing infrastructure, however, currently operates with limited formal linkage to specialist clinical governance. Building on established evidence that hub-and-spoke organization design improves access and continuity in other resource-constrained health systems — including prior applications to Indian rural healthcare more broadly — we propose, as a conceptual framework rather than a system already in operation, a formal Hub-and-Spoke integration in which a Central Government institute such as the Composite Regional Centre (CRC), Kozhikode, would serve as a technical “Hub’ providing task-shared protocol design, tele-rehabilitation supervision, and assistive-technology specification, while the existing LSGI network operates as the ’Spoke.' We identify the conditions under which such integration could plausibly transfer to other settings, and outline a concrete research agenda — spanning feasibility, referral continuity, equity of reach, caregiver cost, workforce competency, and governance — needed before the framework's real-world value can be established.

## 1 Introduction: what is known, and the gap this perspective addresses

Global demand for rehabilitation has escalated alongside population ageing and the rising burden of chronic and neuro-developmental conditions. The WHO estimates that globally about 2.4 billion people currently live with a health condition that may benefit from rehabilitation (1), prompting the 2017 Rehabilitation 2030 initiative's call to integrate these services into Universal Health Coverage rather than treat them as optional (2). Community-based rehabilitation (CBR) has been the WHO's endorsed strategy for extending such services in LMICs since the CBR Guidelines were issued jointly with UNESCO, the ILO, and the International Disability and Development Consortium (3). The evidence base for CBR is real but qualified: a Campbell systematic review found generally positive, if heterogeneous, effects on functioning and quality of life across physical and mental disability groups (4), while subsequent reviews of programme-level evidence have documented recurring implementation problems — stigma, resource shortages, inconsistent monitoring, and, in some cases, a drift toward institutionalization that CBR was designed to avoid. Separately, a substantial task-sharing and task-shifting literature shows that delegating structured rehabilitation activities to trained non-specialist cadres can extend access in primary care settings, though training standardization remains underdeveloped (5).

Centralized specialist institutes, meanwhile, remain essential for complex diagnosis, standardized protocol development, and workforce training, but their population-level reach is constrained by a human-resources-for-health maldistribution that concentrates advanced rehabilitation expertise in urban apex centres (6). For families in rural and semi-urban areas, reaching such centres imposes real and recurring costs — travel time, transport expense, and lost caregiver income — that are consistently identified across LMIC settings as drivers of reduced service uptake and follow-up attrition for children with disabilities (7, 8).

The hub-and-spoke organization design — centralizing complex, technology-intensive services at an anchor “Hub’ while routing routine care through peripheral 'Spoke' sites — is well established in healthcare more broadly, with documented applications in acute stroke networks (9) and, specifically within India, in a rural healthcare access model aimed at making services more affordable, available, and accessible (10). To our knowledge, however, this design has not previously been proposed as a formal framework for linking a national apex disability-rehabilitation institute with an existing local-government-run CBR infrastructure. This is the gap this Perspective addresses.

Kerala offers an unusually developed setting in which to examine this gap. Following the 73rd and 74th Amendments to the Constitution of India, the state's 1996 People's Plan Campaign devolved substantial financial and administrative authority to Local Self-Government Institutions (LSGIs) (11). Working alongside Kudumbashree — a state-wide community network of women originally formed for poverty eradication — LSGIs have, over two decades, come to fund and operate close to 330 BUDS Schools and BUDS Rehabilitation Centres (BRCs) as of 2023 (12), the official Kerala government designation for its neighbourhood day-care and special-education programme for persons with intellectual disability; the term functions as a programme name in the literature rather than a technical acronym, and both BUDS Schools and BRCs are referred to collectively here as “BUDS institutions.’ This local financing occurs against the backdrop of Section 25 of India's Rights of Persons with Disabilities (RPwD) Act 2016, which places a joint statutory duty on the “appropriate Government and the local authorities' to take measures ensuring healthcare access for persons with disabilities (13).

It is important to state plainly, at the outset, what currently exists and what does not. LSGI funding, Kudumbashree household mapping, and the BUDS/BRC network are real, operating arrangements. A formal technical linkage between these local structures and a Central Government specialist institute — the Hub-and-Spoke integration this Perspective proposes — does not yet exist in Kerala or, to our knowledge, elsewhere. What follows is a conceptual framework, illustrated in Table. 1 through the case of the Composite Regional Centre (CRC), Kozhikode, intended to identify how such integration could be built and what would need to be true elsewhere for it to transfer.

**Table 1:** What currently exists in Kerala versus what this framework proposes.

Component	Currently Operating in Kerala	Proposed Hub-and-Spoke Extension (Not Yet Implemented)
Case-finding & household mapping	Kudumbashree Neighbourhood Groups conduct active household-level identification of children and adults with disabilities.	Structured referral pathway routing complex or ambiguous cases from Kudumbashree workers to CRC Kozhikode for specialist assessment.
Day-care, education & vocational support	∼330 LSGI-funded BUDS Schools and BUDS Rehabilitation Centres (BRCs) statewide provide day-care, basic special education and vocational activity.	Structured clinical protocols co-developed with CRC Kozhikode for use by BUDS/BRC staff, with defined competency and supervision standards.
Local financing	LSGIs voluntarily earmark discretionary local-plan funds under general welfare/social-security budget heads to support BUDS institutions.	Joint LSGI–CRC budgeting for assistive-device maintenance and staff training, informed by CRC's technical cost estimates.
Specialist clinical oversight	Available only on-site at CRC Kozhikode and similar apex institutes, requiring travel for families.	Scheduled tele-rehabilitation review sessions between CRC specialists and BUDS/BRC-based staff and caregivers; defined escalation criteria for in-person referral.
Assistive technology	ADIP-scheme camps distribute devices; longitudinal follow-up and maintenance are limited.	Lifecycle model in which CRC issues biomechanical specifications and LSGIs fund fitting, follow-up and local maintenance.

## 2 Architectural pillars of the existing Kerala spoke network

A. The Convergence of Kudumbashree and LSGIs

In partnership with local Panchayats, Kudumbashree Neighbourhood Groups perform active household-level identification of children and adults with disabilities, complementing the limitations of passive hospital-based registries with grassroots visibility (14).

B. BUDS Schools and BUDS Rehabilitation Centres (BRCs)

BUDS Schools (for children with intellectual disabilities under 18) and BRCs (day-care, vocational training, and basic physical maintenance for adults) function as neighbourhood nodes that reduce institutional isolation and allow basic therapeutic routines to be integrated into daily community life (14).

C. Local Financing: Discretionary, Not Currently Mandated

A documented weakness of CBR programmes generally is dependence on volatile external funding. Kerala's model instead relies on local public finance, though it is important to characterize this accurately: qualitative and administrative-data evidence indicates that LSGIs voluntarily earmark funds from their own discretionary revenue — under general budget heads such as 'social welfare,' 'social security,' and “nutrition” — to support BUDS institutions, rather than operating under a specific legally mandated disability-spending quota (14). This discretionary pattern, exercised within the joint healthcare duty that Section 25 of the RPwD Act assigns to local authorities (13), has nonetheless produced sustained, multi-year local investment, though it also means the funding base is not legally guaranteed in the way a fixed statutory quota would be.

## 3 The case for a hub-and-spoke integration

Kerala's Spoke network is well developed for day-to-day socialization, basic maintenance therapy, and case identification, but BUDS/BRC staff generally lack the transdisciplinary expertise to manage complex neuro-developmental conditions, monitor progressive neuromuscular disorders, or fabricate precise assistive technologies. Apex institutes such as CRC Kozhikode hold this expertise — Rehabilitation Council of India (RCI)-approved specialists across Physiotherapy, Occupational Therapy, Speech-Language Pathology, and Prosthetics and Orthotics — but their population-level impact remains limited by the travel and cost barriers already described (6, 7). Consistent with the general benefits documented for hub-and-spoke networks elsewhere in healthcare (9) and specifically within Indian rural health delivery (10), we propose that CRC Kozhikode act as the technical “Hub” and the LSGI network as the 'Spoke,' operationalized across three pillars.

### 3.1 Task-sharing and structured protocol adaptation

We deliberately avoid the term “clinical de-skilling,” used in earlier drafts of this argument, in favour of task-sharing and protocol adaptation — terminology with an established, positive meaning in the health workforce literature (5). Advanced paediatric neuro-rehabilitation methods at CRC Kozhikode would be translated into standardized, competency-based home and centre-based regimens for delivery by trained BUDS/BRC staff, Kudumbashree community workers, and caregivers, consistent with WHO guidance on the rational redistribution of tasks among health workforce teams (15) and with scoping-review evidence that trained community health workers can deliver rehabilitation tasks effectively when training is structured (5).

This pillar raises genuine questions of supervision, scope of practice, and accountability that any implementation would need to resolve rather than assume away. A workable model would require: tiered supervision, with CRC-based RCI-registered professionals retaining named clinical responsibility and periodic direct review of delegated cases; explicit scope-of-practice boundaries defining which tasks (e.g., positioning, basic motor-learning activities, home-programme monitoring) may be delegated and which (e.g., diagnosis, medication-adjacent decisions, complex seating or orthotic prescription) may not; defined escalation criteria triggering referral back to the Hub when a case departs from the expected trajectory; and documentation and accountability protocols recording who delivered what, under whose supervision, consistent with RCI regulatory standards. None of this is yet specified for the Kerala context, and we treat it as a precondition for, not a detail of, any pilot implementation.

### 3.2 Tele-rehabilitation as a supervision and triage channel

A structured digital consultation channel could allow CRC specialists to conduct scheduled reviews with BUDS/BRC-based staff and caregivers, reserving in-person travel to the Hub for acute or complex reviews. We note this is a design proposal, not an evaluated pilot: Kerala's health administration has, separately, reported considering a comparable hub-and-spoke referral model for stroke care, though this remains at a preliminary policy-discussion stage rather than an implemented system (16), and we cite it only as an indication that the state's health administration is receptive to the underlying design logic, not as evidence that tele-rehabilitation hub-and-spoke triage for disability services is already functioning.

### 3.3 Convergent assistive technology provisioning

Passive ADIP-scheme distribution camps without longitudinal follow-up are associated with high device abandonment. We propose a lifecycle-management model in which CRC's Prosthetics, Orthotics, and Physical Rehabilitation specialists generate biomechanical specifications, while Panchayats use local development funds to clear procurement bottlenecks, modify home and school environments, and finance local maintenance — helping ensure devices remain functional as a child grows.

## 4 Conditions for transferability beyond Kerala

Section 5 of this Perspective states plainly that this framework depends on features specific to Kerala and India's constitutional structure. Rather than treat this as a limitation to acknowledge only at the end, we consider directly the more useful question: under what conditions could a comparable integration work elsewhere? At minimum, a transferable setting would likely need (1) a local government tier with genuine fiscal and administrative authority over local welfare spending — not merely an administrative subdivision of central government; (2) an existing grassroots community network, whether government-run or civil-society, capable of household-level case-finding and sustained family engagement; (3) at least one designated specialist institute able to take on Hub-level clinical governance, protocol development, and supervisory responsibility; (4) baseline mobile or internet connectivity sufficient for scheduled tele-supervision; and (5) a legal or policy framework establishing that responsibility for disability healthcare is shared between central/state government and local authorities, as Section 25 of India's RPwD Act does (13), rather than resting solely with one tier. Where these conditions are absent — for instance, in health systems with weak local-government fiscal autonomy or no equivalent of Kudumbashree — the framework's applicability is correspondingly uncertain, and adaptation would need to substitute functionally equivalent local institutions rather than import the Kerala model directly. This framing is offered as testable rather than as a settled claim of transferability.

## 5 Discussion, research agenda, and conclusion

This Perspective has described an existing, decentralized CBR infrastructure in Kerala and proposed — as a conceptual framework, not a system already validated — a formal Hub-and-Spoke integration linking that infrastructure to specialist clinical governance. We have tried to be explicit throughout about which components already operate (LSGI financing, Kudumbashree mapping, the BUDS/BRC network) and which are proposed (task-shared protocols, tele-rehabilitation supervision, assistive-technology lifecycle management, and the governance arrangements these would require).

No empirical data yet exist on patient adherence, cost-effectiveness, or clinical outcomes under a formally integrated Hub-and-Spoke arrangement in Kerala. We propose the following as concrete priorities for the research this framework requires, rather than leaving the future-directions agenda implicit: (1) feasibility and acceptability studies of task-shared protocols among BUDS/BRC staff and caregivers; (2) referral-continuity metrics tracking how reliably cases move between Spoke and Hub when escalation criteria are met; (3) equity-of-reach analysis examining whether the model narrows or widens access gaps across income, distance, and disability type; (4) caregiver cost-burden studies quantifying whether the model measurably reduces travel and lost-income costs relative to the status quo; (5) workforce competency and supervision research establishing minimum training standards and audit mechanisms consistent with RCI norms; (6) assistive-device outcome tracking to test whether lifecycle management reduces abandonment; and (7) governance research examining accountability, liability, and decision rights between Hub and Spoke institutions, which international hub-and-spoke experience identifies as a recurring source of role tension (9).

For global health policymakers working toward the WHO Rehabilitation 2030 agenda, Kerala's existing decentralized infrastructure is a genuine asset, and the Hub-and-Spoke framework proposed here offers one concrete, testable way to connect it to specialist clinical governance. Whether it delivers the anticipated gains in continuity, equity, and cost reduction — in Kerala or elsewhere — is an empirical question this Perspective is intended to motivate, not one it claims to have already answered.

## Data Availability

The original contributions presented in the study are included in the article/Supplementary Material, further inquiries can be directed to the corresponding author.
